# Effect of a distal attachment cuff on adenoma detection rate in screening colonoscopy: Randomized controlled trial in the Spanish population

**DOI:** 10.1055/a-2625-5985

**Published:** 2025-11-03

**Authors:** Aurora Burgos García, Alberto Herreros de Tejada, Carlos Ferre Aracil, Oscar Nogales, Sofía Parejo, Mariana Tavecchia, Belen Agudo, Héctor Julián Canaval Zuleta, Angel Cañete Ruiz, Diego De Frutos Rosa, Julia Arribas Anta, Beatriz Peñas, Rodrigo Borobia, Juan L Mendoza, Cristina Bernardo, Pedro De María, Natalia Lopez Palacios, Mirella Jiménez Gómez, Guillermo Cacho Acosta, Marta Cuadros Martínez, Patricia Mayor Delgado, María Ángeles Ruiz Ramírez, Ana Royuela Vicente, Enrique Rodriguez de Santiago, Consuelo Froilán

**Affiliations:** 1 16268 Gastroenterology Department, Endoscopy Unit, La Paz University Hospital, Madrid, Spain; 2 638528 Hospital La Paz Institute for Health Research, IdiPAZ, Biomedical Research Center in Cancer Network (CIBERONC), Madrid, Spain; 3 16370 Gastroenterology Department, Endoscopy Unit, Puerta de Hierro University Hospital of Majadahonda, Majadahonda, Spain; 4 591905 IDIPHISA, Foundation for Biomedical Research of the Puerta del Hierro University Hospital of Majadahonda, Majadahonda, Spain; 5 16483 Gastroenterology Department, Endoscopy Unit, General University Hospital Gregorio Maranon, Madrid, Spain; 6 16507 Gastroenterology Department, Endoscopy Unit, Ramón y Cajal University Hospital, Madrid, Spain; 7 537482 IRYCIS, Ramón y Cajal Institute for Health Research, Madrid, Spain; 8 16432 Gastroenterology Department, Endoscopy Unit, Alcorcon Hospital Foundation, Alcorcon, Spain; 9 16473 Gastroenterology Department, Endoscopy Unit, 12 de Octubre University Hospital, Madrid, Spain; 10 16267 Gastroenterology Department, Endoscopy Unit, San Carlos Clinic Hospital, Madrid, Spain; 11 16517 Gastroenterology Department, Endoscopy Unit, University Hospital of the Princess, Madrid, Spain; 12 591905 Bioestatistics Unit, Puerta de Hierro University Hospital of Majadahonda, IDIPHISA-CIBERESP-ISCIII, Madrid, Spain; 13 Biomedical Research Network Center in the Thematic Area of Liver and Digestive Diseases (CIBEREHD), Madrid, Spain

**Keywords:** Endoscopy Lower GI Tract, Polyps / adenomas / ..., Colorectal cancer, CRC screening

## Abstract

**Background and study aims:**

Endocuff Vision (EV) is a disposable device designed to improve polyp detection. The primary aim of this study was to evaluate the effect of EV on adenoma detection rate (ADR) and mean number of adenomas per patient (MAP).

**Patients and methods:**

This multicenter randomized controlled trial compared EV-assisted colonoscopy (EAC) and standard colonoscopy (SC). Patients were referred due to a positive fecal immunochemical test (FIT) in a bowel cancer screening program (BCSP), direct screening colonoscopy without prior FIT, surveillance colonoscopy, or family history of colorectal cancer.

**Results:**

In total, 1437 patients (55.9% male; median age, 59 years) were randomized at eight Spanish university hospitals. No significant differences were found in either the ADR (EAC vs. SC, 55.8% vs. 54.3%; *P* = 0.576) or the MAP (1.60 vs. 1.35; *P* = 0.03). Compared with SC, EAC was not associated with a significant improvement in detection rates for advanced adenomas, sessile serrated lesions, or advanced sessile serrated lesions. There was no difference in cecal intubation rate but the successful ileal intubation rate, among patients in whom it was attempted, was lower with EAC (64.3% vs. 86.5% with SC; *P* = 0.001).

**Conclusions:**

EV did not improve either ADR or MAP. EV may increase difficulty of ileal intubation whenever it is attempted.

## Introduction

Colonoscopy is the gold standard for polyp detection and removal and has significantly reduced the incidence of invasive colorectal cancer (CRC) 1 . However, the procedure misses 20% to 26% of adenomas 2 *.*

The adenoma detection rate (ADR) is considered the most crucial quality indicator in CRC prevention because it is inversely associated with CRC-related death 3 . ADR is also an independent predictor of the risk of interval CRC 3 , defined as CRC diagnosed between a negative screening colonoscopy and a scheduled surveillance colonoscopy 3 . The expected ADR of CRC screening programs in Western populations ranges from 20% to 30% 4 when colonoscopy is the initial strategy and exceeds 40% when it is performed in selected populations with positive fecal immunochemical test (FIT) results 5 . However, the ADR is unable to measure the total number of adenomas per individual, leading to the “one and done” phenomenon 6 .

To overcome this limitation and highlight the presence of adenomas, the mean number of adenomas detected per colonoscopy (MAP) has been defined as the total number of adenomas detected divided by the total number of colonoscopies performed 6 . The expected MAP in Western populations remains to be defined, but the MAP of colonoscopies performed for evaluating a positive FIT is significantly higher than the MAP of conventional screening colonoscopy 4 . Therefore, measurement of the MAP in addition to the ADR is suggested in the literature to provide additional information about colonoscopy performance 6 .

To improve the ADR and MAP, several distal attachment devices for colonoscopy have been developed. One of these devices is the Endocuff Vision (EV) (Arc Medical Design Ltd., Leeds, UK), a 2-cm-long flexible polypropylene cap with a single circular row of eight soft projections (Supplementary Fig. 1). It was developed as a relatively simple technique in 2015 as a modification of the first-generation Endocuff (2012), which had eight flexible but shorter and firmer branches arranged in two rows 7 .

Results from the literature are still controversial regarding the impact of EV on adenoma detection and, thus, prevention of CRC development. No studies have evaluated EV in a Spanish asymptomatic screening population with the most common indications for colonoscopy. Accordingly, we designed a multicenter randomized controlled trial (RCT) to compare the ADR and MAP between EV-assisted colonoscopy (EAC) and standard colonoscopy (SC).

## Patients and methods

### Study design

This multicenter, superiority RCT was conducted between June 2018 and April 2019. Patients were recruited from eight tertiary university hospitals in Madrid (Spain). Puerta de Hierro and La Paz University Hospitals were the two coordinating institutions, and the study was led by independent investigators and industry-funded. The funders had no role in study design, data collection, data analysis, data interpretation, or manuscript drafting. To ensure the quality and integrity of the data collected during the study, the Spanish Clinical Research Network (https://scren.eu/) provided external data monitoring services including oversight of the monitoring process, evaluation of its impact on data quality, and comprehensive documentation and reporting.

All 42 participating endoscopists were experienced physicians with between 1 and 20 years’ experience; due to the study design and the high turnover of residents in the participating departments, no residents were involved. The physicians had to have performed at least five procedures with EV prior to patient enrollment. Endoscopist baseline ADR was calculated from the 50 previous colonoscopies performed on a regular basis with indications to the inclusion criteria (Supplementary Table 1). We limited participants with an indication-positive FIT in a bowel cancer screening program (BCSP) to less than 50% of the total enrolled population to avoid a very high baseline ADR, based on prior studies 8 . Endoscopists were stratified into low (≤ 35%), intermediate (36%-49%), high (50%-69%), and very high (≥ 70%) detectors based on their baseline ADR.

The ENDOCOLES study was approved by the Research Ethics Committee of Puerta de Hierro University Hospital and was registered on ClinicalTrials.gov as NCT03436004. Study procedures were performed in accordance with the principles of the Declaration of Helsinki. The study has been reported according to CONSORT guidelines for RCTs 9 . A completed CONSORT Statement checklist is available as supporting information. All patients signed a written informed consent form before study inclusion.

### Patients

**Fig. 1:**
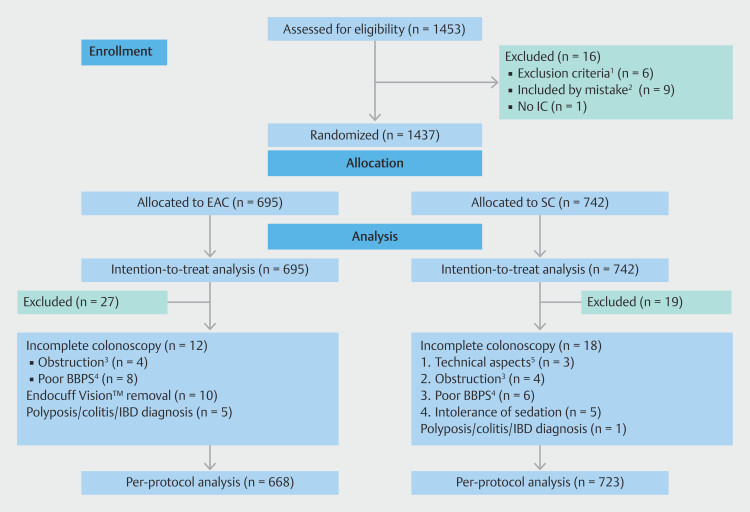
CONsolidated Standards of Reporting Trials (CONSORT) study flow chart 9 for the ENDOCOLES trial. ^1^ Previously unidentified exclusion criteria: inadequate BBPS (a segment score = 0), IBD personal history, prior complete colonoscopy with good visualization of all colonic anatomy (total BBPS ≥6, with all segments score ≥ 2) or previous colectomy. ^2^ Included by mistake: failure in the computer system. ^3^ Obstruction: new cancer diagnosis or colonic stricture. ^4^ Poor BBPS (total BBPS < 6, with a segment score ≤ 1). ^5^ Technical aspects: angulation/fixed sigmoid colon. BBPS, Boston Bowel Preparation Scale; EAC, Endocuff-assisted colonoscopy; IBD, inflammatory bowel disease; IC, informed consent; SC, standard colonoscopy.

Inclusion criteria included patients aged ≥ 18 years referred from a BCSP with a positive FIT, direct screening colonoscopy without prior FIT, surveillance colonoscopy, and family history of CRC (Supplementary Table 1). Clinical and demographic data were collected from all patients. After signing the informed consent form, all participating patients were randomized into two groups: an EAC group and a SC group ( Fig. 1 ).

### Procedure

All colonoscopies were performed with the high-definition (HD) or standard-definition equipment available at the study centers. Patients underwent the procedure under deep sedation by anesthesiologists or non-anesthesiologists according to the standard hospital protocol. Intraprocedural use of carbon dioxide insufflation or conventional air insufflation was also documented. Routine cecal intubation was performed, whereas ileal intubation was attempted only according to the medical criteria of the endoscopist. Time spent on insertion and withdrawal was also recorded. A meticulous mucosal inspection technique during withdrawal was recommended to all participating endoscopists.

Patients received bowel preparation instructions according to the standard hospital protocol. Bowel cleanliness was determined by the endoscopist using the Boston Bowel Preparation Scale. The number, morphology using Paris classification, size, anatomic location, optical diagnosis, and histology of each polyp were documented. NICE classification (used only for Olympus devices with a narrow-band imaging system) was applied for optical diagnosis. All retrieved polyps were sent for histopathologic examination in separate specimen containers.

Adverse events (AEs) during or immediately after the procedure and within a 30-day follow-up period were recorded, including bleeding, perforation, mortality, and other device- or colonoscopy-related complications, such as abdominal pain. Patients were contacted by telephone at between 30 and 45 postprocedure days, and a clinician reviewed the medical history in detail to identify potential complications. A serious AE was defined as one requiring hospitalization; prolongation of a previous hospitalization; new endoscopic, radiological, or surgical treatment; blood transfusion; and/or resulting in death.

### Randomization

Patients were randomized to EAC or SC in a 1:1 ratio using a confidential computer-generated randomization list. This computer system was developed specifically for this trial by an independent biostatistician and then set offline upon study completion. Randomization was stratified by center. The patient was blinded to the allocation, but the endoscopist was not. After entering each patient's allocation data into the computer, the endoscopist could see the randomization arm assigned to each patient.

### Outcomes

The primary outcome was to determine if there was a difference in the ADR and MAP between the EAC and SC groups. Secondary outcomes included determining if there were differences in the remaining variables listed in Supplementary Table 2 between the two groups. The ADR and MAP were also analyzed by colonoscopy indication, endoscopist baseline ADR, use of HD colonoscopy, and maximum polyp size by patient. The safety profile was also documented.

### Sample size and statistical analysis

Sample size calculation was based on the preliminary endoscopist ADR (46% and 35%) and MAP (0.9 and 0.8) data from the two coordinating centers and initial Endocuff studies 7 10 . For a hypothesized 10% increase in the ADR and 30% increase in the MAP (0.3 points between the two groups assuming a standard deviation of 1.9 in a statistically significant manner ( *P* < 0.05) with a power of 80%, it was considered necessary to recruit 631 patients per group, that is, to randomize at least 1262 patients. With an estimated loss rate of 15% (190 patients), at least 1452 patients needed to be recruited.

Descriptive analysis of the sample was performed using absolute and relative frequencies for categorical variables and median and 25th and 75th percentiles or mean and standard deviation for numerical variables, as appropriate, assuming normality. An intention-to-treat (ITT) analysis was performed, including all randomized patients and comparing each group (EAC vs. SC), and was considered the primary analysis of the trial. A per-protocol (PPT) analysis was also conducted. Patients were excluded from the per-protocol analysis in the case of incomplete colonoscopy (technical aspects, bowel obstruction/stenosis, poor bowel preparation, or intolerance to sedation), EV removal, or de novo diagnosis of polyposis, colitis, or inflammatory bowel disease (exclusion criteria).

ADRs were compared between groups using the chi-square test. Binary logistic regression models were used to adjust by center and stratifying by other known factors associated with the ADR, including endoscopist baseline ADR, HD equipment, colonoscopy indication, maximum polyp size by patient, age, and sex. Adjusted odds ratios (aORs) are shown with the corresponding 95% confidence interval (95% CI). Advanced ADR (AADR), serrated lesion detection rate (SDR), and adenoma serrated lesion detection rate (ASDR) were analyzed similarly.

The MAP was compared between groups and adjusted for the center using a Poisson regression model. The adjusted incidence-rate ratio (aIRR) is shown with the corresponding 95% CI. Similar analyses were also conducted stratifying on known factors associated with MAP, including endoscopist baseline ADR, HD equipment, colonoscopy indication, maximum polyp size by patient, age, and sex. Mean advanced adenomas per patient (MAAP), mean serrated lesions per patient (MSP), and mean advanced serrated lesions per patient (MASP) were analyzed similarly.

Other secondary variables (successful cecal intubation rate, EV removal rate, cecal intubation time, withdrawal time, successful ileal intubation rate, and successful rectal and ascending colon retroflexion rate) were analyzed using the Mann-Whitney U test due to a non-normal distribution of the data. Time required for biopsies or polypectomies and bowel cleansing was not included in calculation of withdrawal time. Two-sided *P* < 0.05 was considered to indicate statistically significant differences, except for the primary outcomes, to which the Bonferroni multiple comparison correction was applied. Because there were two primary outcomes, the significance level was corrected to 0.025 for these comparisons.

Additional subanalyses were performed to examine differences in polyp morphology, location, and size between groups using a chi-square, Fisher, or Mann-Whitney U test, depending on type of variable. The 95% CIs and *P* values of secondary analyses were not adjusted for multiplicity and should be considered exploratory. No missing values were detected in the main variables so a complete case analysis was performed.

All analyses were performed using Stata version 16 software for Windows by a biostatistician.

## Results

Between June 2018 and April 2019, 1453 patients were invited to participate in the study; 16 were excluded. Of the 1437 enrolled patients, 695 were assigned to EAC and 742 to SC. All patients were included in the ITT analysis, which was the primary analysis of the trial. After excluding 46 patients post-randomization, the PPT analysis was completed in 1391 patients. The CONSORT trial flow chart 9 is illustrated in Fig. 1 .

Groups were comparable in terms of demographic characteristics, center distribution, and other endoscopic aspects independent of EV ( Table 1 ). Anatomic location, size, morphology, optical diagnosis, and histology of the resected polyps are shown in Table 2 .

**Table:** **Table 1** Baseline demographic characteristics, number of colonoscopies per center, and colonoscopy characteristics.

	EAC (n = 695), %	SC (n = 742), %
**Male sex, n (%)**	400 (57.5%)	404 (54.4%)
**Median age, years (SD)**	59.6 (9.3)	59.1 (9.2)
**ASA classification**		
ASA I	287 (41.3%)	306 (41.3%)
ASA II	339 (48.8%)	383 (51.7%)
ASA III	67 (9.5%)	52 (7%)
ASA IV	2 (0.3%)	0
**Antiplatelet therapy**	48 (6.9%)	60 (8.1%)
**Anticoagulant therapy**	27 (3.9%)	17 (2.3%)
**Prior abdominal surgery**	209 (30%)	234 (31.5%)
**Prior colonoscopy**	249 (35.8%)	265 (35.7%)
**Bowel preparation**		
PEG-ELS	224 (32.2%)	257 (34.7%)
PEG 3350 (low volume)	248 (35.6%)	230 (31%)
Magnesium citrate	222 (31.9%)	254 (34.3%)
Other preparation	2 (0.3%)	0
**Morning procedures**	384 (55.2%)	407 (54.9%)
Split-dosing (morning procedures)	257 (66.9%)	269 (66.1%)
**No. of colonoscopies per center (%)**		
Center 1	157 (22.6%)	158 (21.3%)
Center 2	144 (20.7%)	166 (22.3%)
Center 3	117 (16.8%)	148 (19.9%)
Center 4	76 (10.9%)	60 (8.1%)
Center 5	59 (8.5%)	71 (9.6%)
Center 6	66 (9.5%)	60 (8.1%)
Center 7	51 (7.3%)	51 (6.9%)
Center 8	25 (3.6%)	28 (3.8%)
**Colonoscopy indication**		
BCSP (FIT test+)	395 (56.8%)	427 (57.5%)
Surveillance colonoscopy (adenomas)	128 (18.4%)	124 (16.7%)
Family history of colorectal cancer (low risk)	77 (11.1%)	97 (13.1%)
Family history of colorectal cancer (high risk ^*^ )	65 (9.3%)	70 (9.4%)
Screening colonoscopy (no prior FIT test)	30 (4.3%)	24 (3.2%)
**Anesthesiologist**	403 (58%)	447 (60.2%)
**Use of carbon dioxide**	572 (82.3%)	605 (81.5%)
**High-definition colonoscope**	610 (87.8%)	644 (86.8%)
**BBPS, mean**	8 (7–9)	9 (7–9)
ASA classification, American Society of Anesthesiologists Physical Status classification; BBPS, Boston Bowel Preparation Scale; EAC, Endocuff Vision-assisted colonoscopy; EV, Endocuff Vision; FIT, fecal immunochemical test; PEG, polyethylene glycol; PEG-ELS, polyethylene glycol-electrolyte solution; SC, standard colonoscopy; SD, standard deviation. ^*^ High risk: a first-degree relative with colorectal cancer < 60 years old or ≥ 2 first-degree relative with colorectal cancer. Percentage based on the total number of patients in each group.		

**Table:** **Table 2** Polyp characteristics: location, size, morphology, optical diagnosis, and histology.

	EAC (n = 1750), %	SC (n = 1638), %	*P* value
**Location**			0.21
**Right colon**	**1002 (57.3%)**	**901 (55%)**	
Ileum	0	1 (0.1%)	
Cecum	136 (7.8%)	132 (8.1%)	
Ascending colon	361 (20.6%)	325 (19.8%)	
Hepatic flexure	108 (6.2%)	90 (5.5%)	
Transverse colon	397 (22.7%)	353 (21.5%)	
**Left colon**	**748 (42.7%)**	**737 (45%)**	
Splenic flexure	18 (1%)	22 (1.3%)	
Descending colon	197 (11.3%)	234 (14.3%)	
Sigmoid colon	351 (20.1%)	301 (18.4%)	
Rectum	182 (10.4%)	180 (11%)	
**Size**			0.82
Diminutive (≤ 5 mm)	1136 (64.9%)	1048 (64%)	
Small (6–9 mm)	308 (17.6%)	290 (17,7%)	
Large (≥ 10 mm)	306 (17.5%)	300 (18.3%)	
Morphology ^*^			0.71
**Polypoid**	**1110 (63.4%)**	**1010 (61.7%)**	
Paris 0-Is	905 (51.7%)	811 (49.5%)	
Paris 0-Ip	173 (9.9%)	167 (10.2%)	
Paris 0-Isp	32 (1.8%)	32 (1.9%)	
**Non-polypoid**	**623 (35.6%)**	**611 (37.3%)**	
Paris 0-IIa	532 (30.4%)	509 (31.1%)	
Paris 0-IIb	56 (3.2%)	61 (3.7%)	
Paris 0-IIc	1 (0.06%)	2 (0.1%)	
Homogeneous-type LST-G	12 (0.7%)	18 (1.1%)	
Nodular mixed-type LST-G	5 (0.3%)	4 (0.2%)	
Flat-type LST-NG	12 (0.7%)	16 (1%)	
Pseudodepressed-type LST-NG	12 (0.7%)	16 (1%)	
**Suspicious for invasive carcinoma**	**8 (0.5%)**	**15 (0.9%)**	
**Others**	**9 (0.5%)**	**2 (0.1%)**	
Subepithelial appearance	2 (0.1%)	1 (0.1%)	
Inflammatory appearance	7 (0.4%)	1 (0.1%)	
** Optical diagnosis (NICE ^†^ classification) **	**EAC (n = 1255), %**	**SC (n = 1162), %**	-
Type 1	245 (19.5%)	246 (21.2%)	-
Type 2	995 (79.3%)	896 (77.1%)	-
Type 3	15 (1.2%)	20 (1.7%)	-
**Histology**	**EAC (n = 1658), %**	**SC (n = 1534), %**	***P* value **
Adenomas	1158 (69.8%)	1049 (68.4%)	0.18
Advanced adenomas ^‡^	238 (14.3%)	226 (14.7%)	0.87
Serrated polyps ^§^	233 (14%)	224 (14.6%)	0.76
Advanced serrated polyps ^¶^	31 (1.9%)	27 (1.8%)	0.78
Advanced colorectal neoplasms	19 (1.2%)	31 (2%)	0.15
Non-assessable, negative, or indefinite for neoplasia/dysplasia lesions	237 (14.3%)	222 (14.5%)	0.15
Others	11 (0.7%)	8 (0.5%)	0.15
EAC, Endocuff Vision-assisted colonoscopy; G, granular-type; LST, laterally spreading tumor; n, number of lesions; NG, non-granular-type; SC, standard colonoscopy. *Paris classification. ^†^ NICE classification, Narrow-Band Imaging International Colorectal Endoscopic Classification (Olympus Medical). ^‡^ Advanced adenomas: ≥ 10 mm or high-grade dysplasia. ^§^ Serrated polyps: hyperplastic polyps, sessile serrated lesions, and traditional serrated lesions. ^¶^ Advanced serrated polyps: ≥ 10 mm or dysplasia.			

Endoscopists were stratified into low (≤ 35%), intermediate (36%-49%), high (50%-69%), and very high (≥ 70%) detectors according to their previously calculated baseline ADR. Percentages of endoscopists in each group were 9.7%, 30.6%, 38.9%, and 20.7%, respectively. Our global ADR was 54% and more than 90% of the endoscopists had a baseline ADR exceeding 35%.

### Adenoma detection rate

ADR was similar in both groups in both ITT analysis (EAC vs. SC, 55.8% vs. 54.3% [aOR 0.94, 95% CI 0.76–1.16, *P* = 0.576]) and PPT analysis (EAC vs. SC, 57% vs. 55.5% [aOR 0.93, 95% CI 0.75–1.16, *P* = 0.542]) ( Table 3 ). No differences were found when the ADR analysis was also adjusted for hospital, colonoscopy indication, endoscopist baseline ADR, use of HD equipment, and maximum lesion size by patient (Supplementary Table 3).

**Table:** **Table 3** Primary outcome: ADR and MAP.

	Analysis	EAC (n = 695), %	SC (n = 742), %	aOR	95% CI	*P* value
**ADR**	ITT	388 (55.8%)	403 (54.3%)	0.94	0.76–1.16	0.576
	PPT	381 (57%)	401 (55.5%)	0.93	0.75–1.16	0.542
	**Analysis**	**EAC (n = 695)**	**SC (n = 742)**	**aIRR**	**95% CI**	***P* value **
**MAP**	ITT	1.66	1.41	0.85	(0.73–0.98)	0.029
	PPT	1.70	1.44	0.84	(0.73–0.98)	0.030
ADR, adenoma detection rate; aIRR, adjusted incidence-rate ratio by the stratification variable “Hospital”; aOR, adjusted odds ratio by the stratification variable “Hospital”; EAC, Endocuff Vision-assisted colonoscopy; ITT, intention-to-treat analysis; MAP, mean number of adenomas per patient; PPT, per-protocol analysis; SC, standard colonoscopy.						

### Mean number of adenomas per patient

Compared with SC, EV was not associated with a significantly higher MAP in both ITT analysis (EAC vs. SC, 1.60 vs. 1.35 [aIRR 0.85, 95% CI 0.73–0.98, *P* = 0.029]) and PPT analysis (EAC vs. SC, 1.63 vs. 1.38 [aIRR 0.85, 95% CI 0.73–0.98, *P* = 0.030]).

Additional factors influencing differences in the MAP between the two groups were also examined. The MAP was slightly higher in BCSP patients with a positive FIT in both ITT analysis (EAC vs. SC, 2.15 vs. 1.80 [IRR in the SC group 0.83, 95% CI 0.69–0.99, *P* = 0.04]) and PPT analysis (EAC vs. SC, 2.20 vs. 1.83 [IRR in the SC group 0.83, 95% CI 0.69–0.99, *P* = 0.04]) ( Table 3 ). The MAP was also significantly higher in certain subgroups in both ITT and PPT analyses: high-detector endoscopists (baseline ADR 50%-69%) and use of HD equipment (Supplementary Table 4).

### Advanced adenomas and serrated lesions

When evaluating advanced adenomas, AADR was also similar in both groups in ITT analysis (EAC vs. SC, 22.4% vs. 21.6% [95% CI 0.74–1.22, *P* = 0.56]) and PPT analysis (EAC vs. SC, 22.7% vs. 21.8% [95% CI 0.74–1.22, *P* = 0.69]). For serrated lesions, no differences in SDR and ASDR were found, even after additional adjustment for hospital, colonoscopy indication, endoscopist baseline ADR, use of HD equipment, and polyp size (Supplementary Table 5).

No differences were observed between groups in MAAP and MSP (Supplementary Table 6).

### Colonoscopy outcomes directly related to EV

No significant differences were found between groups in successful cecal intubation rate (> 97% in both groups), cecal intubation time (mean of 4 minutes in both groups), withdrawal time (9–10 minutes in both groups), and effect of EV on rectal and ascending colon retroflexion (similar in both groups).

Thirty patients, without differences between groups, had an incomplete colonoscopy for the reasons listed in Table 4 . The elective EV removal rate was 1.4% (10 patients). The cecal pole was eventually reached in nine of these 10 patients. In only one case, the examination could not be completed due to patient anthropometric characteristics. No cases of involuntary EV loss were documented. However, the successful ileal intubation rate was significantly different between the EAC (64.3%) and SC (86.5%) groups ( *P* < 0.001), with a similar intubation attempt rate in both groups (EAC vs. SC, 42.3% vs. 45.0%, *P* = 0.3). The odds ratio (EAC vs. SC) was 0.28 (95% CI 0.19–0.41, *P* = 0.001).

**Table:** **Table 4** Colonoscopy technical outcomes stratified by group.

	EAC (n = 695), %	SC (n = 742), %	*P* value
Successful cecal intubation rate	683 (98.3%)	724 (97.6%)	0.58
**Cecal intubation failure (causes):**			0.30
Technical aspects (angulation/fixed sigmoid colon)	0	3 (0.4%)	
Obstruction (neoplasia, colonic stricture, inguinal hernia)	4 (0.6%)	4 (0.5%)	
Poor BBPS ^*^	8 (1.1%)	6 (0.8%)	
Intolerance of sedation	0	5 (0.7%)	
Median cecal intubation time, minutes (SD)	4 (3–6)	4 (3–6)	0.49
Attempted intubation of the ileum ^†^	294 (42.3%)	334 (45%)	0.30
Successful ileal intubation rate ^‡^	189 (64.3%)	289 (86.5%)	**0.001**
Median withdrawal time, minutes (SD)	10 (7–12)	9 (7–11)	0.32
Attempted ascending colon retroflexion ^§^	81 (11.6%)	83 (11.2%)	0.78
Successful ascending colon retroflexion rate	81 (100%)	83 (100%)	0.78
Attempted rectal retroflexion ^¶^	465 (66.9%)	516 (69.5%)	0.28
Rectal retroflexion rate	465 (100%)	516 (100%)	0.28
EV voluntary removal rate	11 (1.4%)	--	--
EV involuntary loss rate	0	--	--
EAC, Endocuff Vision-assisted colonoscopy; EV, Endocuff Vision; SC, standard colonoscopy. *Poor BBPS (Boston Bowel Preparation Scale): total BBPS < 6, with a segment score ≤ 1. ^†^ Attempted intubation of the ileum: Total number of attempted intubations of the terminal ileum in all patients. ^‡^ Successful ileal intubation rate: Total number of successful ileal intubations divided by the total number of colonoscopies in which ileal intubation had been attempted in each group. ^§¶^ Total number of patients in whom ascending colon and rectum retroflexion was performed: retroflexion was possible in all of these patients.			

### Adverse events

AEs were documented in 39 patients (2.7%), with no differences between the EAC and SC groups regarding type of complication, severity, time of onset, need for hospitalization, or duration of the event ( Table 5 ).

**Table:** **Table 5** Adverse events.

	EAC (n = 695), %	SC (n = 742), %	*P* value
**Total number of adverse events**	**22 (3.2%)**	**17 (2.3%)**	**0.40**
**Lower gastrointestinal bleeding**	6 (0.9%)	4 (0.5%)	0.46
Severe lower gastrointestinal bleeding*	4 (0.6%)	1 (0.1%)	0.16
Endoscopic treatment	4 (0.6%)	0	0.14
Admission	2	1	0.61
**Gastrointestinal perforation**	**0**	**2 (0.3%)**	**0.17**
Severe gastrointestinal perforation*	0	2 (0.3%)	0.17
Surgical treatment	0	1 (0.1%)	0.33
Admission	0	2 (0.3%)	0.50
**Death**	**0**	**0**	**0**
**Other adverse events**	**3 (0.4%)**	**2 (0.3%)**	**0.29**
Abdominal pain	2 (0.3%)	1 (0.1%)	0.61
Hemorrhoid pain	0	1 (0.1%)	1.00
Fever	1 (0.1%)	0	0.48
EAC, Endocuff Vision-assisted colonoscopy; SC, standard colonoscopy. *A severe adverse event was considered to have occurred if the patient required hospitalization; prolongation of a previous hospitalization; new endoscopic, radiological, or surgical treatment; blood transfusion; and/or death occurred.			

## Discussion

### Summary of main results

In this multicenter RCT, we evaluated the EV device in an asymptomatic Spanish population with the most representative indications for colonoscopy. A total of 1437 patients were enrolled, making this one of the largest studies comparing the device with SC. Our results indicated no differences in either the ADR or the MAP between EAC and SC. An overview of previous studies of Endocuff or EV is shown in Supplementary Table 7.

### Interpretation of results

Several studies of EV have shown conflicting results regarding the ADR. First-generation 10 11 12 13 and second-generation 14 15 16 17 Endocuff studies showed ADR improvements ranging from 4.7% to 17%. In contrast, studies by Van Doorn et al. 18 , Bhattacharyya et al. 19 , Jaensch et al. 20 , Von Figura et al. 21 , and Rex et al. 22 found no significant increase in the ADR. A systematic review by Aziz et al. 23 also found no significant improvement in ADR compared with HD colonoscopy.

A possible explanation for these findings is that studies reporting ADR improvements with Endocuff 10 11 12 13 14 15 16 17 often had a low control-arm ADR (< 41%). Our high control-arm ADR (54%) was similar to that in studies where no benefit was observed, suggesting that reported improvements in some studies might reflect initially poor polyp detection rates. This could be due to endoscopist quality or experience or the type of population included 12 . For example, certain geographic areas, such as Latin America, may have a lower prevalence of adenomas 12 .

In addition, several meta-analyses 24 25 26 27 28 suggested that Endocuff may benefit operators with a low ADR (< 30%-35%) more than those with a high ADR (>35%), but our study did not support that finding.

Consistent with most studies 17 18 22 24 29 30 , no significant difference was found between EAC and SC in AADR, SDR, and ASDR. The ItaVision Study 15 showed a 21% increase in AADR only in BCSP patients with a positive FIT. The ADENOMA study 16 found a small increase in SDR, but its clinical significance was questionable. Another RCT of serrated polyposis 31 also showed no significant differences.

In our study, EV did not improve MAP compared with SC. Several RCTs showed a MAP improvement with EV 15 16 17 18 , particularly in BCSP patients with a positive FIT 15 16 . We also identified a slight increase in this last group of patients, but the difference between EAC and SC was modest (0.25, *P* < 0.003). Furthermore, this finding was exploratory because it was obtained from subanalyses, which could make the clinical relevance of this result debatable. Future studies could be useful to further evaluate the impact of EV on MAP and its correlation with CRC incidence, particularly in BCSP patients with a positive FIT.

No significant differences were found between EAC and SC in MAAP, MSP, and MSAP. Even though Endocuff was reported to assist in detection of smaller lesions in previous studies 10 11 13 14 18 29 32 , we did not find differences in MAP according to maximum lesion size by patient, anatomical location 15 30 32 33 34 , or morphology 15 19 32 .

Use of EV improved MAP in operators with a high ADR (50%-69%) and HD devices, but the clinical significance of these findings should, again, be interpreted cautiously because this was a secondary subgroup analysis. HD device results in the literature are heterogeneous, because some studies did not collect endoscope model information 16 22 .

Successful cecal intubation rate, cecal intubation time, withdrawal time, and successful rectal retroflexion were not affected by EV. EV removal rate was low at 1.4%; most colonoscopies were completed even when EV was removed. No involuntary loss of the EV was reported. However, differences in ileal intubation rates between groups were observed. Ileal intubation was only attempted according to the medical criteria of the endoscopist. Among patients in whom ileal intubation was attempted (42.3% EAC vs. 45% SC; *P* = 0.30), there was some degree of difficulty in ileal intubation with the EV device (64.3% EAC vs. 86.5% SC; *P* < 0.001). In addition, several endoscopists subjectively reported that the EV might hinder easy access to the ileum when bending the colonoscope due to the protruding rubber arms. This finding is consistent with those of other studies 7 12 14 21 , although many did not collect such information 15 16 17 18 19 22 32 and the conclusions remain unclear.

Studies with the first-generation Endocuff reported minor mucosal tears 7 11 25 . The second-generation EV appears to have addressed these issues 16 19 , and we found no AEs attributable to EV, such as perforation or bleeding.

### Strengths and limitations

A significant strength of this study is the multicenter, randomized design involving eight university hospitals, which has obtained a large and representative sample of the Spanish population. This is the first study conducted in our geographical area with such broad colonoscopy indications, including CRC screening within a BCSP. Endoscopists with varying baseline ADRs performed the procedures after completing a training period with the EV, which enhances the generalizability of our findings.

Limitations include lack of blinding of the endoscopists, because the rubber arms of the EV are visible to the physician during colonoscopy. Another limitation was inability to calculate adenoma or serrated polyp loss rates, because this was not a tandem study. Although tandem studies are useful, they increase procedure risk, reduce patient enrollment, and could create a nonrealistic condition 13 . Although our global ADR was 54%, the sample size calculation was based on lower baseline ADRs, indicating that a potentially larger sample size might have been needed. Finally, all 42 participating endoscopists were experienced physicians, with between 1 and 20 years of practice. Although baseline ADRs varied among them, the proportion of low detectors (ADR ≤ 35%) was relatively low. This is likely attributable to the fact that the participating centers were actively involved in CRC screening programs and had a strong focus on endoscopic diagnosis training. Due to the study design, trainee residents were not included and that could be noted as a limitation.

### Clinical implications

Our findings suggest that EV does not improve either the ADR, which aligns with the results of several large RCTs with high control-arm ADRs 18 19 21 , or the MAP. The MAP was slightly higher in BCSP patients with a positive FIT, but the clinical relevance of this finding might be questioned, as it was secondary subgroup analysis. Based on our findings, we cannot recommend the systematic addition of EV to diagnostic colonoscopy but it should be tailored to the specific conditions of each center and endoscopist.

## Conclusions

In conclusion, our results showed no significant difference in either the ADR or the MAP. Although EV is safe and feasible and does not increase procedure time, it may hamper ileal intubation in some cases. Future studies are necessary to further evaluate the impact of EV on the MAP and its correlation with CRC incidence.

## Supplementary Material

Supplementary Material Zusatzmaterial

Supplementary Material Zusatzmaterial
