# Supplementary material for: Effect of a distal attachment cuff on adenoma detection rate in screening colonoscopy: Randomized controlled trial in the Spanish population
Source: Endosc Int Open. 2025 Nov 3;13:a26255985. doi: 10.1055/a-2625-5985 (PMC12599166; doi:10.1055/a-2625-5985)
Supplement: Supplementary file 1 — Supplementary Material [file 10-1055-a-2625-5985_26299099.pdf]

Supplementary Fig. 1 "ARV120" Endocuff Vision (EV) device. The EV is a 2-cm-long, flexible cap with one circular row of eight soft projections. The device attaches to the end of the colonoscope. Personal photographs by author.

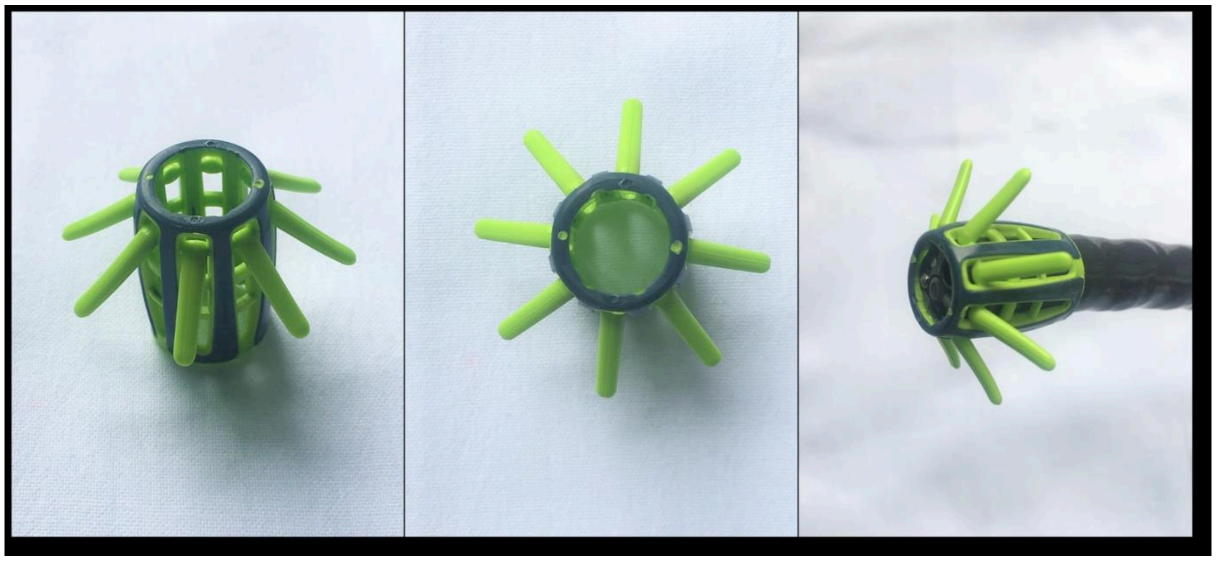

Supplementary Table 1 Inclusion and exclusion criteria.

Inclusion criteria	Exclusion criteria
Patients aged ≥ 18 years	Absolute contraindication to colonoscopy<br>Intestinal obstruction<br>Intestinal perforation<br>Acute diverticulitis
BCSP (FIT+)	Digestive symptoms
Surveillance colonoscopy (adenomas)	Previous (total or partial) colonic resection
Family history of CRC	Personal or family history of inherited syndrome of CRC or polyposis syndromes
Screening colonoscopy without prior FIT	Personal history of CRC or IBD
	Previous colonoscopy within last year with adequate bowel preparation (BBPS ≥ 6, at least ≥ 2 in each colon segment)
	Pregnancy or lactation period
	Express desire of the patient or significant medical, psychological, psychiatric, geographic, or social problem that could interfere with the study

Endosc Int Open | DOI 10.1055/a-2625-5985 | © The Author(s).

BBPS, Boston Bowel Preparation Score; BCSP, bowel cancer screening program; CRC, colorectal cancer; FIT, fecal immunochemical test; IBD, inflammatory bowel disease.

Endosc Int Open | DOI 10.1055/a-2625-5985 | © The Author(s).

Supplementary Table 2 Main variables and definitions.

Variable	Acronym	Definition
Adenoma detection rate	ADR	Total number of colonoscopies in which one or more histologically confirmed adenomas are found divided by the total number of colonoscopies performed in each group
Mean number of adenomas detected per patient	MAP	Total number of adenomas divided by the total number of colonoscopies performed in each group
Advanced adenoma detection rate	AADR	Total number of colonoscopies in which one or more advanced adenomas (high-grade dysplasia or ≥ 10 mm) are found divided by the total number of colonoscopies performed in each group
Serrated lesion detection rate	SDR	Total number of colonoscopies in which one or more serrated lesions (hyperplastic polyp, sessile serrated lesion, or traditional serrated lesions) are found divided by the total number of colonoscopies performed in each group
Advanced serrated lesion detection rate	ASDR	Total number of colonoscopies in which one or more advanced serrated lesions (dysplasia or ≥ 10 mm) are found divided by the total number of colonoscopies performed in each group
Mean number of advanced adenomas detected per patient	MAAP	Total number of advanced adenomas (high-grade dysplasia or ≥ 10 mm) divided by the total number of colonoscopies performed in each group
Mean number of serrated lesions detected per patient	MSP	Total number of serrated lesions (hyperplastic polyps, sessile serrated lesions, or traditional serrated lesions) divided by the total number of colonoscopies performed in each group
Mean number of advanced serrated lesions detected per patient	MASP	Total number of advanced serrated lesions (dysplasia or ≥ 10 mm) divided by the total number of colonoscopies performed in each group
Successful cecal intubation rate	--	Total number of complete colonoscopies (determined by visualization of the ileocecal valve and the appendiceal orifice) divided by the total number of colonoscopies performed in each group

Endosc Int Open | DOI 10.1055/a-2625-5985 | © The Author(s).

Cecal intubation time	--	Mean time (minutes) to reach the cecal pole divided by the total number of colonoscopies in each group
Successful ileal intubation rate	--	Total number of successful ileal intubations divided by the total number of colonoscopies in which ileal intubation was attempted in each group
Withdrawal time	--	Mean time (minutes) from the cecal pole to the anal margin (excluding time taken for polypectomies and bowel cleaning) divided by the total number of colonoscopies performed in each group
Successful ascending colon retroflexion rate	--	Total number of successful ascending colon retroflexions divided by the total number of colonoscopies in which retroflexion was attempted in each group
Successful rectal retroflexion rate	--	Total number of successful rectal retroflexions divided by the total number of colonoscopies in which retroflexion was attempted in each group
EV voluntary removal rate	--	Total number of colonoscopies in which EV had to be removed because cecal intubation was not possible divided by the total number of colonoscopies in each group
EV involuntary loss rate	--	Total number of colonoscopies in which EV was involuntarily lost divided by the total number of colonoscopies in each group

EV, Endocuff Vision.

Endosc Int Open | DOI 10.1055/a-2625-5985 | © The Author(s).

Supplementary Table 3 Adenoma detection rate (ADR) in intention-to-treat and per-protocol analyses according to colonoscopy indication, endoscopist baseline ADR, high-definition equipment, and maximum lesion size by patient

ADR	Analysis	EAC (n = 695)%	SC (n = 742)%	OR	95% CI	*P* value
**ADR according to colonoscopy indication**						
BCSP with positive FIT	ITT	257 (65.1%)	268 (62.8%)	0.90	0.68-1.20	0.49
	PPT	250 (66.7%)	266 (63.9%)	0.88	0.66-1.19	0.42
Surveillance colonoscopy	ITT	77 (60.2%)	68 (54.8%)	0.80	0.49-1.33	0.39
	PPT	77 (60.6%)	68 (55.7%)	0.81	0.49-1.35	0.43
Family history of CRC	ITT	43 (30.3%)	57 (34.1%)	1.19	0.74-1.93	0.47
	PPT	43 (30.7%)	57 (35.4%)	1.23	0.76-2.00	0.39
Screening colonoscopy without prior FIT	ITT	11 (36.67%)	10 (41.67%)	1.23	0.41-3.71	0.71
	PPT	11 (42.3%)	10 (41.67%)	0.97	0.32-2.99	0.96
**ADR according to endoscopist's baseline ADR**						
Low (≤35%)	ITT	23 (34.3%)	32 (43.8%)	1.49	0.75-2.96	0.25
	PPT	23 (36.5%)	32 (43.8%)	1.35	0.68-2.70	0.38
Intermediate (36%-49%)	ITT	141 (61%)	112 (53.6%)	0.74	0.50-1.08	0.11
	PPT	140 (61.7%)	111 (55.8%)	0.78	0.53-1.15	0.22
High (50%-69%)	ITT	142 (55%)	163 (54.1%)	0.96	0.69-1.35	0.83
	PPT	140 (55.8%)	162 (54.5%)	0.95	0.68-1.33	0.77
Very high (≥70%)	ITT	82 (59%)	96 (60.4%)	1.06	0.67-1.68	0.80
	PPT	78 (61.4%)	96 (62.3%)	1.04	0.64-1.69	0.87

Endosc Int Open | DOI 10.1055/a-2625-5985 | © The Author(s).

**ADR according to HD equipment**						
Without HD equipment	ITT	38 (44.7%)	52 (53%)	0.89	0.78-2.50	0.25
	PPT	38 (45.8%)	52 (55.3%)	0.88	0.70-1.10	0.2
With HD equipment	ITT	350 (57.4%)	350 (54.5%)	1.40	0.71-1.11	0.31
	PPT	343 (58.6%)	348 (55.4%)	1.46	0.81-2.65	0.26
**ADR according to maximum lesion size**						
Diminutive (≤5 mm)	ITT	140 (64.5%)	156 (71.2%)	1.36	0.90-2.04	0.13
	PPT	139 (64.9%)	156 (71.2%)	1.34	0.89-2.00	0.16
Small (6-9 mm)	ITT	82 (82%)	70 (81.4%)	0.96	0.45-2.02	0.91
	PPT	81 (81.8%)	70 (82.4%)	1.04	0.49-2.21	0.92
Large (≥ 10 mm)	ITT	166 (88.8%)	177 (87.2%)	0.86	0.47-1.59	0.63
	PPT	161 (89.4)	175 (87.1%)	0.79	0.42-1.49	0.47

ADR, adenoma detection rate; BCSP, bowel cancer screening program; CRC, colorectal cancer; EAC, Endocuff Vision-assisted colonoscopy; FIT, fecal immunochemical test; HD, high-definition; ITT, intention-to-treat analysis; OR, odds ratio; PPT, per-protocol analysis; SC, standard colonoscopy.

Endosc Int Open | DOI 10.1055/a-2625-5985 | © The Author(s).

Supplementary Table 4 Mean number of adenomas per patient in intention-to-treat and per-protocol analyses according to colonoscopy indication, endoscopist's baseline adenoma detection rate, high-definition equipment, and maximum lesion size per patient.

MAP	Analysis	EAC (n = 695)	SC (n = 742)	IRR	95% CI	*P* value
**MAP according to colonoscopy indication**						
BCSP with positive FIT	ITT	2.15	1.80	0.83	(0.69-0.99)	0.04
	PPT	2.20	1.83	0.83	(0.69-0.99)	0.04
Surveillance colonoscopy	ITT	1.72	1.22	0.71	(0.51-1.00)	0.05
	PPT	1.73	1.24	0.72	(0.51-1.01)	0.06
Family history of CRC	ITT	0.48	0.63	1.30	(0.85-1.98)	0.21
	PPT	0.49	0.66	1.33	(0.00-2.03)	0.17
Screening colonoscopy without prior FIT	ITT	0.63	1	1.57	(0.70-3.54)	0.27
	PPT	0.73	1	1.37	(0.62-3.03)	0.44
**MAP according to endoscopist baseline ADR**						
Low (≤ 35%)	ITT	1.10	1	0.90	(0.47-1.75)	0.76
	PPT	1.17	1	0.85	(0.44-1.64)	0.63
Intermediate (36%-49%)	ITT	1.59	1.36	0.86	(0.66-1.12)	0.24
	PPT	1.61	1.42	0.88	(0.68-1.14)	0.35
High (50%-69%)	ITT	1.78	1.37	0.77	(0.60-0.99)	0.04
	PPT	1.80	1.39	0.77	(0.59-0.99)	0.04
Very high (≥ 70%)	ITT	1.85	1.75	0.95	(0.70-1.29)	0.72

Endosc Int Open | DOI 10.1055/a-2625-5985 | © The Author(s).

	PPT	1.92	1.80	0.94	(0.69-1.28)	0.69
**According to HD equipment**						
Without HD equipment	ITT	0.92	1.33	1.44	(0.85-2.44)	0.17
	PPT	0.93	1.38	1.47	(0.87-2.49)	0.15
With HD equipment	ITT	1.77	1.42	0.81	(0.69-0.94)	0.01
	PPT	1.81	1.46	0.81	(0.69-0.94)	0.01
**According to maximum lesion size per patient**						
Diminutive (≤ 5 mm)	ITT	1.27	1.17	0.92	(0.76-1.13)	0.44
	PPT	1.27	1.17	0.92	(0.76-1.12)	0.43
Small (6-9 mm)	ITT	2.40	2.3	0.94	(0.72-1.22)	0.64
	PPT	2.41	2.28	0.94	(0.73-1.23)	0.68
Large (≥ 10 mm)	ITT	3.44	2.95	0.86	(0.71-1.03)	0.11
	PPT	3.47	2.96	0.85	(0.71-1.03)	0.10

BCSP, bowel cancer screening program; CRC, colorectal cancer; ADR, adenoma detection rate; EAC, Endocuff Vision-assisted colonoscopy; FIT, fecal immunochemical test; HD, high-definition; IRR, incidence-rate ratio; ITT, intention-to-treat analysis; MAP, mean number of adenomas per patient; PPT, per-protocol analysis; SC, standard colonoscopy.

Endosc Int Open | DOI 10.1055/a-2625-5985 | © The Author(s).

Supplementary Table 5 Advanced adenoma detection rate, serrated lesion detection rate, and advanced serrated lesion detection rate in intention-to-treat and per-protocol analyses

	Analysis	EAC (n = 695)	SC (n = 742)	OR	95% CI	*P* value
AADR	ITT	156 (22.4%)	160 (21.6%)	0.95	0.74-1.22	0.69
	PPT	152 (22.7%)	158 (21.8%)	0.95	0.74-1.22	0.69
SDR	ITT	148 (21.3%)	137 (18.5%)	0.84	0.64-1.08	0.18
	PPT	143 (21.4%)	137 (18.9%)	0.86	0.66-1.11	0.25
ASDR	ITT	21 (3%)	21 (2.8%)	0.93	0.50-1.73	0.83
	PPT	19 (2.8%)	21 (2.9%)	1.02	0.54-1.91	0.94

AADR, advanced adenoma detection rate; ASDR, advanced serrated lesion detection rate; EAC, Endocuff Vision-assisted colonoscopy; ITT, intention-to-treat analysis; OR, odds ratio; PTT, per-protocol analysis; SC, standard colonoscopy; SDR, serrated lesion detection rate.

Endosc Int Open | DOI 10.1055/a-2625-5985 | © The Author(s).

Supplementary Table 6 Mean number of advanced adenomas per patient, mean number of serrated lesions per patient, and mean number of advanced serrated lesions per patient in intention-to-treat and per-protocol analyses

	Analysis	EAC (n = 695)	SC (n = 742)	IRR (SC group)	95% CI	*P* value
MAAP	ITT	0.34	0.30	0.89	(0.69-1.14)	0.35
	PPT	0.34	0.31	0.89	(0.70-1.15)	0.39
MSP	ITT	0.33	0.30	0.90	(0.68-1.18)	0.45
	PPT	0.33	0.31	0.95	(0.72-1.24)	0.70
MASP	ITT	0.44	0.36	0.81	(0.41-1.61)	0.56
	PPT	0.39	0.37	0.96	(0.47-1.95)	0.90

EAC, Endocuff Vision-assisted colonoscopy; IRR, incidence-rate ratio; ITT, intention-to-treat analysis; MAAP, mean number of advanced adenomas detected per patient; MASP, mean number of advanced serrated lesions detected per patient; MSP, mean number of serrated lesions detected per patient; PTT, per-protocol analysis; SC, standard colonoscopy.

Endosc Int Open | DOI 10.1055/a-2625-5985 | © The Author(s).

Supplementary Table 7 Overview of previous studies of Endocuff or Endocuff Vision and the current study.

Study	Country	Study design	No. centers	No. endoscopists	No. patients	Colonoscopy indication	Device	EAC ADR (%)	SC ADR (%)	Absolute difference (%)	*P* value	EAC MAP (median ± SD or median [IQR])	SC MAP (median ± SD or median [IQR])	*P* value
**Floer et al., 2014**	Germany	Parallel group	4	10	492	Screening, surveillance, or diagnosis	EC	35.4%	20.7	14.7	0.0001	1 (1-2)	1 (1-3)	0.851
**Biecker et al., 2015**	Germany	Parallel group	2	6	498	Screening, surveillance, or diagnosis	EC	36	28	8	0.043	2 (1-3)	1 (1-2)	0.002
**Van Doorn et al, 2017**	The Netherlands	Parallel group	5	20	1063	Screening, surveillance, or diagnosis	EC	52	52	0	NS	1.36 ± 2.10	1.17 ± 1.65	0.08
**González-Fernández et al., 2017**	Mexico	Parallel group	1	18	337	Screening	EC	22.4	13.5	8.9	0.02	0.3 ± 0.25	0.21 ± 0.26	0.02
**De Palma et al., 2018**	Italy	Tandem	a	4	288	Screening, surveillance, or diagnosis	EC	29.6	26.3	3.3	0.002	0.63	0.52 (NR)	NR
**Wada et al., 2018**	Japan	Parallel group	2	1	447	Screening, surveillance, or diagnosis	EC	52.5	39.2	15	0.0002	1.11±1.41	0.66 ± 0.99	<0.01
**Cavallaro et al., 2018**	Italy	Retrospective	1	NR	546	Screening or surveillance	EC	52.4	47.4	5	0.1	1.11 ± 1.55	0.87 ± 1.24	0.02
**Bhattacharyya et al. 2017**	United Kingdom	Parallel group	1	4	530	Screening	EV	60.9	63	2.1	NS	1.3 ± 1.8	1.4 ± 1.5	0.54
**Ngu et al., 2019**	United Kingdom	Parallel group	7	>70	1772	Screening, surveillance, or diagnosis	EV	40.9	36.2	4.7	0.02	0.95 ± 1.89	0.75 ± 1.40	0.02
**Jacob et al., 2019**	Australia	Parallel group	1	6	282	Mixed population (all patients)	EV	36.8	28.9	7.9	NR	NR	NR	NR
**Rex et al., 2020**	USA	Parallel group	2	2	200	Screening or surveillance	EV	61.4	52.5	8.9	0.206	1.43 ± 1.79	1.07 ± 1.62	0.07
**Karsenti et al., 2020**	France	Cluster-randomized crossover trial	1	22	2058	Screening, surveillance, or diagnosis	EV	39.2	29.4	9.8	0.001	0.78 ± 1.32	0.54 ± 1.10	<0.001

Endosc Int Open | DOI 10.1055/a-2625-5985 | © The Author(s).

**Von Figura et al., 2020**	Germany	Parallel group	1	14	250	Mixed population (all patients)	EV	42.6	38.1	4.5	NS	1.4	1.7	0.93
**Zorzi et al., 2020**	Italy	Parallel group	13	25	1864	Screening	EV	47.8	40.8	7	CI+	0.94	0.77	0.001
**Zimmermann-Fraedrich et al., 2022**	Germany	Parallel group	9	23	1416	Screening	EV	39.5	32.2	7.3	0.004	0.57	0.51	0.045
**Jaensch et al., 2022**	Denmark	Parallel group	1	36	1166	Screening	EV	59.2	60.5	1.3	0.01	NR	NR	NR
**Burgos et al., 2024**	Spain	Parallel group	8	42	1437	Screening and surveillance	EV	55.8	54.3	1.5	0.576	1.66	1.41	0.03

ADR, adenoma detection rate; MAP, mean number of adenomas per patient; CI+, confidence interval mean significant difference; EAC, Endocuff-assisted colonoscopy; EC, Endocuff (first generation); EV, Endocuff Vision (second generation); IQR, interquartile range; NR, not reported; NS, not significant; SC, standard colonoscopy; SD, standard deviation.

Endosc Int Open | DOI 10.1055/a-2625-5985 | © The Author(s).
